# The impact of antiretroviral treatment on mortality trends of HIV-positive adults in rural Uganda: a longitudinal population-based study, 1999–2009

**DOI:** 10.1111/j.1365-3156.2012.02841.x

**Published:** 2012-07-30

**Authors:** Ivan Kasamba, Kathy Baisley, Billy N Mayanja, Dermot Maher, Heiner Grosskurth

**Affiliations:** 1 MRC/UVRI Uganda Research Unit on AIDSEntebbe, Uganda; 2 London School of Hygiene and Tropical MedicineLondon, UK

**Keywords:** antiretroviral therapy, mortality, HIV+, Masaka, Uganda

## Abstract

**Objective:**

To investigate trends in all-cause adult mortality after the roll-out of an antiretroviral therapy (ART) programme in rural Uganda.

**Methods:**

Longitudinal population-based cohort study of approximately 20 000 residents in rural Uganda. Mortality in adults aged 15–59 years was determined for the 5-year period (1999–2003) before introduction of ART in January 2004 and for the 5-year period afterwards. Poisson regression was used to estimate mortality rate ratios (RRs) for the period before ART, 1 year after ART introduction (from January 2004 to January 2005) and more than 1 year after ART introduction. Trends in mortality were analysed by HIV status, age and sex.

**Results:**

Before ART became available, the mortality rate (deaths per 1000 person-years) was 4.0 (95% CI = 3.3–4.8) among HIV-negative individuals and 116.4 (95% CI = 101.9–133.0) among HIV-positive individuals. During the period January 2004–end November 2009, 279 individuals accessed ART. In the year after ART was introduced, the mortality rate (deaths per 1000 person-years) among HIV-negative individuals did not change significantly (adjusted RR = 0.95, 95% CI = 0.61–1.47), but among HIV-positive individuals dropped by 25% to 87.4 (adjusted RR = 0.75, 95% CI = 0.53–1.06). In the period 2005–2009, the mortality rate (deaths per 1000 person-years) among HIV-positive individuals fell further to 39.9 (adjusted RR = 0.33, 95% CI = 0.26–0.43). The effect was greatest among individuals aged 30–44 years, and trends were similar in men and women.

**Conclusion:**

The substantially reduced mortality rate among HIV-positive individuals after ART roll-out lends further support to the intensification of efforts to ensure universal access to ART.

## Introduction

Antiretroviral therapy (ART) has reduced HIV-related morbidity and mortality in both developed (Mocroft *et al.* 2003) and developing countries (Jahn *et al.* 2008). However, treatment coverage for HIV-infected patients must be provided and maintained on a sufficient scale for individual-level health improvements to be translated into population-level reductions in mortality. The need is particularly urgent in sub-Saharan Africa, which bears the brunt of the global HIV epidemic with an estimated 1.3 million HIV-related deaths in 2009 (72% of global HIV-related mortality) (UNAIDS 2010).

Since 2003, there has been a rapid scale up of ART for HIV-infected individuals in poor countries (UNAIDS/WHO 2009). One of the important measures of successful large-scale ART programmes is the effect on population-level mortality. Monitoring the effectiveness of ART at population level helps governments make informed decisions about investments in ART programmes and aids in estimations of the numbers of people with HIV infection who will need health care in future. Very few studies from sub-Saharan Africa on the population-level effect of ART on mortality have been published. Recent studies in Malawi (Floyd *et al.* 2010) and South Africa (Herbst *et al.* 2009) have demonstrated a reduction in population mortality shortly after introduction of ART. However, there have been no studies yet of the population-level impact of ART stratified by individuals’ HIV status.

Population-based cohort studies provide data on HIV-related mortality that are more representative of the general population than data from clinic-based studies, which provide information only on the morbidity and mortality of individuals who have accessed health care. An open population-based cohort established in rural Uganda in 1989 provided the opportunity to investigate the effect of ART introduction in 2004 on adult mortality.

## Methods

### Setting

Uganda has been recovering since 1986 from previous civil, political and economic turmoil. The population is approximately 30 million, annual gross national income is $300 per capita and mean life expectancy at birth is 50 years (UNICEF 2008). Uganda is one of the countries in Africa where the HIV epidemic was first reported and which was initially very severely affected by HIV/AIDS. Peak national HIV seroprevalence was 18% in 1992, with a subsequent 70% decline through the 1990s until reaching a plateau of about 6% at the end of that decade (Kirungi *et al.* 2006; UNAIDS 2006).

A general population cohort was established in 1989 in rural south-west Uganda for the purposes of HIV surveillance and now comprises approximately 20 000 residents of 25 neighbouring villages in Masaka district, not far from Lake Victoria. There are no tarmac roads, and access may be difficult during the rains. The community comprises mostly people belonging to the Baganda tribe, with 15% of Rwandese origin. Religious affiliation is mostly Christian, with a significant Muslim minority (28%). Half the population is under 15 years of age. The vast majority of dwellings are distributed throughout the countryside rather than clustered in villages, which represent administrative areas demarcated on maps rather than population centres. Study participants are mostly subsistence farmers. The main income-earning activities are growing bananas, coffee and beans, and trading in produce and fish (Nakibinge *et al.* 2009). HIV prevalence in the study area declined from 8.5% in 1990 to 6.2% in 2000 but thereafter rose to 7.7% in 2005 (Shafer *et al.* 2008).

### Annual population-based cohort HIV survey

Full details of the cohort and annual HIV serosurvey have been published elsewhere (Kengeya-Kayondo *et al.* 1996; Mbulaiteye *et al.* 2002). In brief, an annual household surveys have been conducted since 1989, with all study village residents eligible for inclusion. Community sensitisation activities precede each survey round, including local council briefings and village meetings. All households are visited by teams, which undertake, in turn, mapping, census (recording births, deaths and in- and out-migrants) and survey. Consenting residents aged at least 3 years are interviewed at home in the local language by trained survey staff and provide a blood sample for HIV testing. Survey participants who indicate that they would like to receive their HIV test result are referred to local voluntary counselling and testing (VCT) centres, where they are informed of their result and counselled.

### ART programme in the study clinic

In January 2004, ART became available in the study clinic. Eligibility is determined according to Uganda Ministry of Health criteria: CD4 cell count of 200 cells/μl (250 cells/μl if pregnant) or below, WHO clinical stage 4 disease or advanced stage 3 disease with persistent or recurrent oral thrush or invasive bacterial infections regardless of CD4 count (Ministry of Health, Republic of Uganda 2003). Cotrimoxazole prophylaxis is given according to the Ministry of Health guidelines (Ministry of Health, Republic of Uganda 2005). Other HIV-positive individuals from the population cohort who present at the study clinic with symptoms and signs of HIV infection, and those identified through attendance at the local VCT centres, are also offered ART care if required and if they accept are also enrolled in the clinical cohort.

By the end of 2008, overall ART coverage in the programme was 69% (Kazooba *et al.* 2012). As the research clinic is the only ART provider in the locality, it is likely that only very few ART-eligible individuals from the study population access ART from other providers. Eligible patients are intensively prepared through three counselling visits and a medical examination before starting ART and thereafter are followed-up regularly at the clinic. First-line treatment consists of a combination of zidovudine (AZT), lamivudine (3TC) and nevirapine (NVP), with a possibility of switching to stavudine (d4T) in case of AZT toxicity and to efavirenz in case of NVP toxicity or concurrent tuberculosis treatment.

### Statistical methods

We assessed overall mortality rates in all adults aged 15–59 years in the general population cohort over the 10-year period from 1999 to 2009. We chose to focus on this age range as it is mostly affected by HIV-related mortality. Person-time was defined from the date the individual was first seen in the census or was aged 15 years, whichever was later, until the earliest of date of death, migration out of the area, last seen in the census or age 60. Multiple episodes of observation and gaps were allowed, if individuals moved out of the surveillance area and later returned. Gaps of <2 years were ignored.

Mortality rates were compared between three periods: during the 5 years before the start of the national roll-out in January 2004, during the 1 year after ART become available in the area (from January 2004 until January 2005, denoted ART period 1) and during the 4-year period from January 2005 until end 2009, denoted ART period 2.

To assess mortality by HIV status, data were left-truncated at the date of the first HIV test. Seroconverters contributed deaths and person-years at risk to the HIV-infected population from date of their first positive HIV test. They also contributed person-years at risk to the HIV-negative population before their last negative test. Survival was evaluated using Kaplan–Meier curves. Poisson regression was used to estimate rate ratios (RR) for mortality, stratified by age group and HIV status. Age-stratified mortality RR were adjusted for sex to allow for possible changes in the sex ratio over time.

### Ethical considerations

The study received ethical approval from the Science and Ethics Committee of the Uganda Virus Research Institute and the Uganda National Council of Science and Technology. Use of unique identifying numbers ensured participants’ confidentiality.

## Results

Between 1999 and November 2009, we recorded 686 deaths in 18 077 individuals aged 15–59 years during 76 989 person-years of observation, with a crude death rate of 8.9 deaths per 1000 person-years (95% CI 8.3–9.6; Table 1). Among the 18 077 individuals, 1333 (7.4%) were known to be HIV-positive, including 476 who seroconverted during the period of observation. Of the 65 674 person-years of observation in persons of known HIV status, 4446 person-years (6.8%) were contributed by HIV-positive individuals.

**Table 1:** Number of individuals, observed deaths, person-years of follow-up and crude death rates

Adults 15–59, 1999–2009	Male	Female	All
Total individuals	8414	9663	18 077
Seroconverters	199	277	476
Seroprevalent	279	578	857
Uninfected	6568	7515	14 083
HIV status unknown (never tested)	1368	1293	2661
Person-years of observation			
All adults*	36 723	40 266	76 989
HIV uninfected†	29 158	32 070	61 228
HIV positive†	1768	2678	4446
HIV status unknown (never tested)‡	3397	2385	5782
Deaths	328	358	686
Seroconverters	47	57	104
Seroprevalent	106	130	236
Uninfected	124	114	238
HIV status unknown (never tested)	51	57	108
Mortality rate/1000 person-years (95% CI)			
All adults	8.9 (8.0–10.0)	8.9 (8.0–9.9)	8.9 (8.3–9.6)
HIV uninfected	4.3 (3.6–5.1)	3.6 (3.0–4.3)	3.9 (3.4–4.4)
HIV positive	86.5 (73.8–101.4)	69.8 (60.5–80.6)	76.5 (68.8–85.1)
HIV status unknown (never tested)	15.0 (11.4–19.8)	23.9 (18.4–31.0)	18.7 (15.5–22.6)

* Total person years calculated from the date first seen in census or aged 15 years, whichever was later, until earliest of date of out-migration, last seen in the census or age 60. Includes the period before the date of the first HIV test, among those tested.

† Data left truncated at date of first HIV test (person-years between date first seen in census and first HIV test are ignored).

‡ Person years calculated as described in *, among individuals who never had an HIV test.

**Figure 1:**
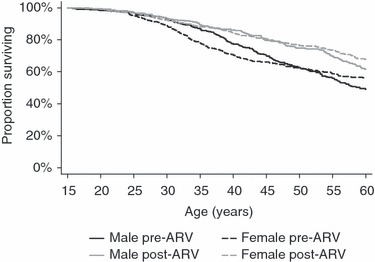
Kaplan–Meier survival function for the entire study population in the 5 years before (1999–2003) and after (2004–2009) antiretroviral therapy introduction, by sex.

Figure 1 shows survival probability between the ages of 15–60 years for the entire population, by sex, in the 5 years before ART became available and in the 5-year period after the introduction of ART in 2004. Before 2004, the probability of dying from any cause between the ages of 15 and 60 years was 51% for men (95% CI = 45–57) and 44% for women (95% CI = 39–49). During 2004–2009, it reduced to 38% (95% CI = 33–44) for men and 32% (95% CI = 28–37) for women. Mortality was significantly lower in 2005–2009 (ART period 2) than in the 5 years before ART introduction (in men, age-adjusted RR = 0.60, 95% CI = 0.47–0.76; in women, age-adjusted RR = 0.57, 95% CI = 0.44–0.71; Table 2). Furthermore, there was a non-significant reduction in mortality in the first year after ART became available (ART period 1; in men, age-adjusted RR = 0.84, 95% CI = 0.58–1.20; in women, age-adjusted RR = 0.81, 95% CI = 0.57–1.15; Table 2). In contrast, among individuals aged 60 years and older, overall mortality did not change (age- and sex-adjusted RR = 1.01, 95% CI = 0.76–1.36 in ART period 1 compared with pre-ART and RR = 1.00, 95% CI = 0.84–1.20 in ART period 2; data not shown).

**Table 2:** All-cause mortality in adults aged 15–59 years, pre- and post-ART roll-out*

	Adults 15–59, 1999–2009, entire population		
	
	Deaths/pyrs (rate/1000 pyr)	Unadjusted RR (95% CI)	Adjusted RR† (95% CI)
All individuals		*P* < 0.001	*P* < 0.001
0–5 years pre ART	390/35 626 (10.9)	1	1
ART period 1‡	71/7713 (9.2)	0.84 (0.65–1.08)	0.82 (0.64–1.06)
ART period 2‡	225/33 650 (6.7)	0.61 (0.52–0.72)	0.58 (0.50–0.68)
Stratified by sex			
Male		*P* < 0.001	*P* < 0.001
0–5 years pre ART	185/17 145 (10.8)	1	1
ART period 1‡	34/3671 (9.3)	0.86 (0.60–1.24)	0.84 (0.58–1.20)
ART period 2‡	109/15 907 (6.9)	0.64 (0.50–0.80)	0.60 (0.47–0.76)
Female		*P* < 0.001	*P* < 0.001
0–5 years pre ART	205/18 481 (11.1)	1	1
ART period 1‡	37/4042 (9.2)	0.83 (0.58–1.17)	0.81 (0.57–1.15)
ART period 2‡	116/17 743 (6.5)	0.59 (0.47–0.74)	0.57 (0.45–0.71)
Stratified by age			
15–29 years		*P* = 0.06	*P* = 0.06
0–5 years pre ART	109/20 447 (5.3)	1	1
ART period 1‡	21/4259 (4.9)	0.92 (0.58–1.48)	0.92 (0.58–1.47)
ART period 2‡	67/17 980 (3.7)	0.70 (0.52–0.95)	0.70 (0.51–0.94)
30–44 years		*P* < 0.001	*P* < 0.001
0–5 years pre ART	192/9853 (19.5)	1	1
ART period 1‡	34/2268 (15.0)	0.77 (0.53–1.11)	0.77 (0.53–1.11)
ART period 2‡	84/10 046 (8.4)	0.43 (0.33–0.55)	0.43 (0.33–0.55)
45–59 years		*P* = 0.29	*P* = 0.29
0–5 years pre ART	89/5327 (16.7)	1	1
ART period 1‡	16/1185 (13.5)	0.81 (0.47–1.38)	0.81 (0.47–1.38)
ART period 2‡	74/5624 (13.2)	0.79 (0.58–1.07)	0.79 (0.58–1.07)

ART, antiretroviral therapy; RRs, rate ratios.

* ART roll-out defined as 1 January 2004.

† Adjusted for sex and age (RRs for all individuals), age (RRs stratified by sex), or sex (RRs stratified by age).

‡ ART period 1 defined as the first year after ART roll-out (1 January 2004 to 1 January 2005). ART period 2 defined as the 4-year period from 1 January 2004 to 31 December 2009).

§ All estimates include participants whose HIV status was unknown (never tested).

**Figure 2:**
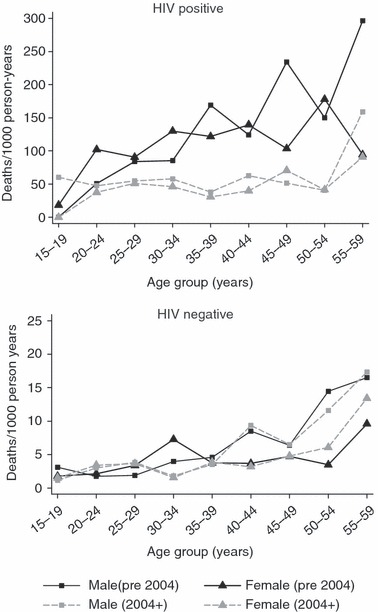
Mortality rates by sex and age-group, in the 5 years before antiretroviral therapy (ART) introduction and after ART roll-out in 2004, among HIV-positive (top) and HIV-negative (bottom) adults aged 15–59 years.

Among the 325 individuals of known HIV status who died in the 5-year period before ART introduction, 216 (66.4%) were HIV infected. Mortality rates by HIV status are shown in Figure 2 and Table 3. In the 5 years before ART became available, mortality (deaths per 1000 person-years) was 4.0 (95% CI = 3.3–4.8) among HIV-negative individuals and 116.4 (95% CI = 101.9–133.0) among HIV-positive individuals. During this period, the estimated population-attributable fraction (PAF) of HIV in adults aged 15–59 years was 64%. In the year after ART was introduced (ART period 1), the mortality rate (deaths per 1000 person-years) among HIV-negative individuals was not significantly different (adjusted RR = 0.95, CI = 0.61–1.47) but had shrunk by 25% to 87.4 (adjusted RR = 0.75, CI = 0.53–1.06) among HIV-positive adults. In the period 2005–2009 (ART period 2), mortality among HIV-negative individuals did not change significantly (adjusted RR = 0.93, CI = 0.71–1.21); however, among HIV-positive individuals, it fell further to 39.9 deaths per 1000 person-years (adjusted RR = 0.33, CI = 0.26–0.43). During the 5-year period after ART introduction (2004–2009), the estimated PAF of HIV had dropped to 45%. Among HIV-negative individuals, there was no evidence of a difference in mortality trends between age groups or between men and women (test for interaction *P* = 0.55 and *P* = 0.96, respectively). Among HIV-positive individuals, in the year after ART introduction, the reduction in mortality was greatest among those aged 45–59 years; thereafter, the impact was greatest among individuals aged 30–44 years (*P* = 0.14, test for interaction). There was no evidence of a difference in mortality trends between men and women (*P* = 0.76, test for interaction).

**Table 3:** All-cause mortality in adults aged 15–59 years from 1999 to 2009, pre- and post-ART roll-out*, by HIV status

	HIV negative		HIV positive	
		
	Deaths/pyrs (rate/1000 pyr)	Adjusted RR† (95% CI)	Deaths/pyrs (rate/1000 pyr)	Adjusted RR† (95% CI)
All individuals				
Time relative to ART roll-out		*P* = 0.86		*P* < 0.001
0–5 years pre ART	109/27 402 (4.0)	1	216/1855 (116.4)	1
ART period 1‡	24/6306 (3.8)	0.95 (0.61–1.47)	38/435 (87.4)	0.75 (0.53–1.06)
ART period 2‡	105/27 519 (3.8)	0.93 (0.71–1.21)	86/2156 (39.9)	0.33 (0.26–0.43)
Stratified by sex				
Male		*P* = 0.82		*P* < 0.001
0–5 years pre ART	58/13 100 (4.4)	1	98/780 (125.6)	1
ART period 1‡	12/2992 (4.0)	0.89 (0.48–1.66)	16/174 (92.0)	0.73 (0.43–1.24)
ART period 2‡	54/13 066 (4.1)	0.89 (0.62–1.29)	39/814 (47.9)	0.36 (0.25–0.52)
Female		*P* = 0.98		*P* < 0.001
0–5 years pre ART	51/14 302 (3.6)	1	118/1075 (109.8)	1
ART period 1‡	12/3315 (3.6)	1.01 (0.54–1.89)	22/261 (84.3)	0.77 (0.49–1.21)
ART period 2‡	51/14 453 (3.5)	0.97 (0.66–1.43)	47/1342 (35.0)	0.31 (0.22–0.43)
Stratified by age				
15–29 years		*P* = 0.99		*P* = 0.004
0–5 years pre ART	38/16 138 (2.4)	1	52/641 (81.1)	1
ART period 1‡	8/3601 (2.2)	0.94 (0.44–2.02)	10/141 (71.0)	0.88 (0.44–1.72)
ART period 2‡	36/15 237 (2.4)	1.00 (0.64–1.58)	21/578 (36.3)	0.45 (0.27–0.74)
30–44 years		*P* = 0.27		*P* < 0.001
0–5 years pre ART	36/6994 (5.1)	1	121/964 (125.5)	1
ART period 1‡	5/1701 (2.9)	0.57 (0.22–1.46)	25/241 (103.8)	0.83 (0.54–1.28)
ART period 2‡	28/7641 (3.7)	0.71 (0.43–1.16)	39/1185 (32.9)	0.26 (0.18–0.38)
45–59 years		*P* = 0.72		*P* < 0.001
0–5 years pre ART	35/4270 (8.2)	1	43/250 (171.9)	1
ART period 1‡	11/1004 (11.0)	1.34 (0.68–2.63)	3/53 (56.4)	0.33 (0.10–1.05)
ART period 2‡	41/4641 (8.8)	1.08 (0.69–1.69)	26/393 (66.1)	0.39 (0.24–0.63)

ART, antiretroviral therapy; RRs, rate ratios.

* ART roll-out defined as 1 January 2004.

† Adjusted for sex and age (RRs for all individuals), age (RRs stratified by sex), or sex (RRs stratified by age).

‡ ART period 1 defined as the first year after ART roll-out (1 January 2004 to 1 January 2005). ART period 2 defined as the 4-year period from 1 January 2004 to 31 December 2009).

There was a 27-fold increased mortality risk in HIV-positive compared with HIV-negative individuals aged 15–59 before ART introduction, which fell after ART introduction to 21-fold in the first year (ART period 1; adjusted RR = 21.5, 95% CI = 12.9–36.0) and ninefold in the subsequent 4 years (ART period 2; adjusted RR 9.4, 95% CI = 7.0–12.5) (Table 4). The trends of falling mortality risk after ART introduction were similar in each age group (Table 4).

**Table 4:** Comparison of mortality rates by HIV status in adults aged 15–59 years, pre- and post-ART roll-out, by age group

	Age group (years)							
	
	15–29		30–44		45–59		15–59	
				
	Deaths/pyrs (rate/1000 pyr)	RR* (95% CI)	Deaths/pyrs (rate/1000 pyr)	RR* (95% CI)	Deaths/pyrs (rate/1000 pyr)	RR* (95% CI)	Deaths/pyrs (rate/1000 pyr)	RR† (95% CI)
0–5 years pre ART								
HIV negative	38/16 138 (2.4)	1	36/6994 (5.1)	1	35/4270 (8.2)	1	109/27 402 (4.0)	1
HIV positive	52/641 (81.1)	34.1 (22.3–52.2)	121/964 (125.5)	24.3 (16.7–35.2)	43/250 (171.9)	20.1 (12.9–31.5)	216/1855 (116.4)	27.1 (21.4–34.2)
ART period 1‡								
HIV negative	8/3601 (2.2)	1	5/1701 (2.9)	1	11/1004 (11.0)	1	24/6306 (3.8)	1
HIV positive	10/141 (71.0)	31.7 (12.5–80.5)	25/241 (103.8)	35.3 (13.5–92.2)	3/53 (56.4)	4.9 (1.4–17.6)	38/435 (87.4)	21.5 (12.9–36.0)
ART period 2‡								
HIV negative	36/15 237 (2.4)	1	28/7641 (3.7)	1	41/4641 (8.8)	1	105/27 519 (3.8)	1
HIV positive	21/578 (36.3)	15.2 (8.8–26.3)	39/1185 (32.9)	9.0 (5.6–14.7)	26/393 (66.1)	7.3 (4.4–11.9)	86/2156 (39.9)	9.4 (7.0–12.5)

ART, antiretroviral therapy; RRs, rate ratios.

* Adjusted for sex and interaction between HIV status and time period. *P*-value for interaction = 0.06 in those aged 15–29, *P* = 0.002 in those 30–34 and *P* = 0.003 in those aged 45–59.

† Adjusted for age, sex and interaction between HIV status and time period.

‡ ART period 1 defined as the first year after ART roll-out (1 January 2004 to 1 January 2005). ART period 2 defined as the 4-year period from 1 January 2004 to 31 December 2009).

## Discussion

Our results show the considerable effect of HIV on mortality at the population level and the substantial reversal of this effect by introduction of ART. During the 5-year period after ART introduction in 2004, overall mortality in adults decreased by 32% in men and 37% in women. Although there was some reduction in overall adult mortality in the first year after ART introduction, the largest effect was seen in the subsequent 4 years. Our findings indicate that, among adults aged 15–59 years, the PAF of HIV in the 5 years before ART introduction was 64% and had fallen to 45% in the 5 years after ART was introduced.

The non-significant reduction in mortality in the first year after ART introduction may be the result of several factors. When ART was first introduced, many of those starting therapy may have entered the program with more advanced disease and lower CD4 counts than the current thresholds for ART initiation (CD4 < 250), and thus mortality may have been higher. ART uptake in 2004 may have been lower than in subsequent years, because fewer people knew their HIV-positive status. From 2004 to 2006, only 46% of HIV-positive persons knew their status, while in 2008, 56% knew their status (Kazooba *et al.* 2012).

A recent study in South Africa found a significant drop in standardised all-cause mortality in adults aged 25–49 years in the 2 years after ART introduction: 16% in men and 8% in women (Herbst *et al.* 2009). In a study in rural northern Malawi in which ART scale-up started in 2005 and reached 70% coverage in 2008, all-cause mortality rate among 15–59 year olds fell by 32% (Floyd *et al.* 2010). However, HIV prevalence in both cohorts (21.5% in South Africa and 11.4% in Malawi) is higher than in our study cohort. The reduction in mortality in Malawi was greater in the area near the main roads and thus closer to the ART clinic. Estimated ART coverage in our cohort in 2008, at 69% (Kazooba *et al*. 2012), is similar to that in the Malawi study but lower than coverage in the South Africa study area (estimated at 84% in 2006).

When separated by HIV status, we found no evidence of change in mortality among the HIV-negative adults but a significant reduction among HIV-positive adults. Thus, the drop in mortality we observed at the population level is explained by fewer deaths among HIV-positive persons. From 2004 onwards, HIV-positive participants in the clinical cohort received cotrimoxazole in addition to ART. As cotrimoxazole reduces mortality (Mermin *et al.* 2004, World Health Organization 2006a, b), the marked reduction in mortality among HIV-positive adults in our study population may reflect the availability and uptake of both cotrimoxazole and ART.

Using the previous WHO guidelines, in which the CD4 cell count threshold for ART initiation was 200 cells/mm^3^ (World Health Organization 2006a, b), estimated national ART coverage in Uganda in 2007 was 33% (UNAIDS 2008). In our cohort, 48% of the HIV-positive adults were estimated to need ART in 2008, and overall estimated ART coverage, as a proportion of the estimated number in need of treatment was 69% (Kazooba *et al.* 2012). This is higher than the national coverage but far from ideal. While in principle all ART-eligible individuals had access to ART, not all of them made use of the opportunity, partly because they did not ask to be tested, partly because some did not want to be enrolled on ART.

Our estimated mortality rates in this study differ from the previous reports of mortality in this setting, based on a clinical cohort drawn from the general population cohort (Van der Paal *et al.* 2007). This is in part owing to differences in the composition of the two groups, in particular the older age distribution of HIV-negative individuals in the clinical cohort.

A strength of this study is that the annual population cohort survey collects demographic data, including information on dates of birth, death and in- and out-migration, allowing us precise denominators to calculate mortality rates. Dates of HIV infection can also be estimated reasonably well because of the annual serosurvey. A limitation of the study is that average annual serosurvey participation is about 60–65%, although a much higher percentage has ever participated. For example, although only 65% of residents aged 13 years and older participated in the 2008–2009 serosurvey, 84% had ever participated in a previous year. Based on the current mortality in individuals who have never been tested for HIV (12.7 per 1000 person-years in 2005–2009), we estimate around 26% of those who have never participated to be HIV positive.

Despite encouraging VCT for HIV among all persons in the general population cohort and referral of people with HIV infection to the study clinic, substantial mortality remains among HIV-positive individuals. Although mortality among HIV-positive adults has fallen significantly since the introduction of ART, it remains much higher than mortality among HIV-negative adults, particularly in the younger age groups. Before HIV-related mortality falls to levels seen in developed countries, a much larger proportion of HIV-positive persons will need to find out their HIV serostatus and be referred early to obtain care at an earlier stage of HIV infection. In addition to benefits for individuals, greater proportions of people who know their HIV status early and of people with HIV infection who start ART early may confer population-level benefits through lowered viral loads, less infectivity and reduced HIV transmission (Garnett & Baggaley 2009).
